# Targeting reactive oxygen species and fat acid oxidation for the modulation of tumor-associated macrophages: a narrative review

**DOI:** 10.3389/fimmu.2023.1224443

**Published:** 2023-07-21

**Authors:** Yujian Teng, Licheng Xu, Wenjing Li, Pengyan Liu, Linli Tian, Ming Liu

**Affiliations:** Department of Otolaryngology, The Second Affiliated Hospital of Harbin Medical University, Harbin, Heilongjiang, China

**Keywords:** tumor-associated macrophages, reactive oxygen species, fatty acid oxidation, tumor microenvironment, recruitment, polarization, antioxidant systems, metabolism

## Abstract

Tumor-associated macrophages (TAMs) are significant immunocytes infiltrating the tumor microenvironment(TME). Recent research has shown that TAMs exhibit diversity in terms of their phenotype, function, time, and spatial distribution, which allows for further classification of TAM subtypes. The metabolic efficiency of fatty acid oxidation (FAO) varies among TAM subtypes. FAO is closely linked to the production of reactive oxygen species (ROS), which play a role in processes such as oxidative stress. Current evidence demonstrates that FAO and ROS can influence TAMs’ recruitment, polarization, and phagocytosis ability either individually or in combination, thereby impacting tumor progression. But the specific mechanisms associated with these relationships still require further investigation. We will review the current status of research on the relationship between TAMs and tumor development from three aspects: ROS and TAMs, FAO and TAMs, and the interconnectedness of FAO, ROS, and TAMs.

## 1 Introduction

Various components of the tumor microenvironment(TME) play a crucial role in tumorigenesis and progression. Among these components, tumor-associated macrophages (TAMs) can regulate the TME through different infiltration levels and polarization characteristics (1, 2). TAMs in TME mainly originate from peripheral blood mononuclear cells (PBMCs) and tissue-resident macrophages (TRMs) (3). PBMCs are recruited to TME primarily mediated by chemokine-mediated pathways involving CCR2/CCL2 (4, 5), CSF1/M-CSF (6–8), and others. TRMs are present in tissues during embryonic development with tissue-specific and self-renewal abilities. In the past, TAMs were often classified into two distinct phenotypes, M1 and M2, with lipopolysaccharides and interferon-gamma (IFN-gamma) or interleukin (IL)-4 and IL-13 as representative inducers, respectively (9). M1 promotes the formation of an inflammatory environment and possess tumor-killing capabilities. M2 assists in constructing an immunosuppressive microenvironment by secreting IL-10, transforming growth factor beta (TGF-beta), or prostaglandin E2 (PGE2) to promote tissue repair and tumorigenic development. However, researchers have revealed that in most cases, TAMs exhibit a transitional phenotype between M1 and M2 (10–12). The emergence of this transitional subtype has expanded the previous classification criteria for TAMs. It has been demonstrated that TAMs can regulate tumor progression by modulating their own phenotype (13–15).

**Figure 1:**
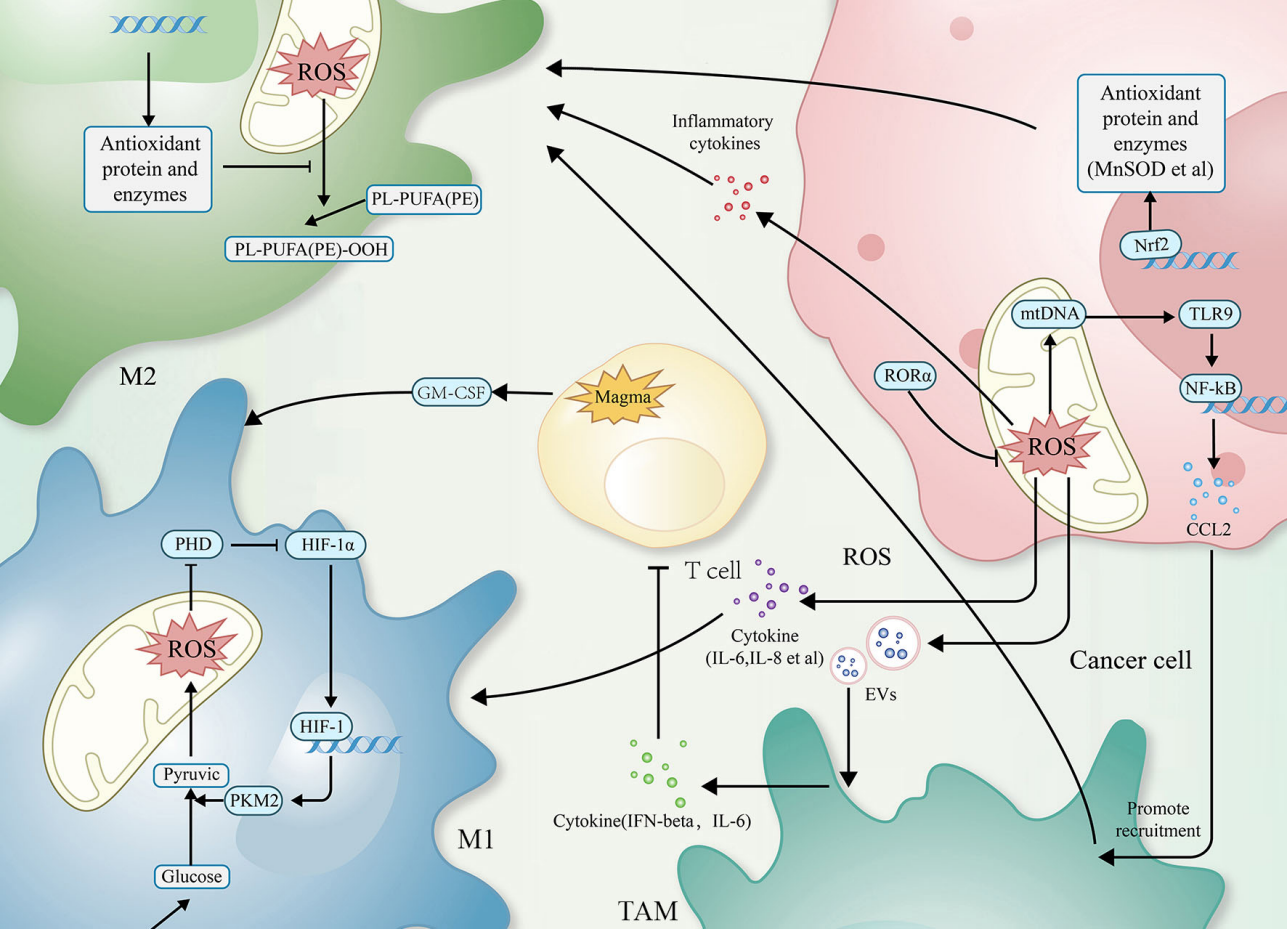
The relationship between ROS and TAMs. Simplified figure depicting various possible mechanisms of TAMs associated with ROS at a cellular-level affect oncogenesis and development of tumor in TME.

Reactive oxygen species (ROS), as a kind of redox byproduct, mainly includes superoxide, hydrogen peroxide (H2O2), and hydroxyl radical (HO-) (16). Mitochondria, endoplasmic reticulum,and peroxisomes are the primary sites of ROS production (17). ROS possess high chemical reactivity, and the oxidative stress effects will occur when the cell’s antioxidant capacity cannot coordinate the excess ROS (18, 19). For tumors, such ROS and oxidative stress effect are both necessary and lethal, and they regulate TAM-related mechanisms through multiple signaling pathways (20) (as shown in **Figure 1**).

**Figure 2:**
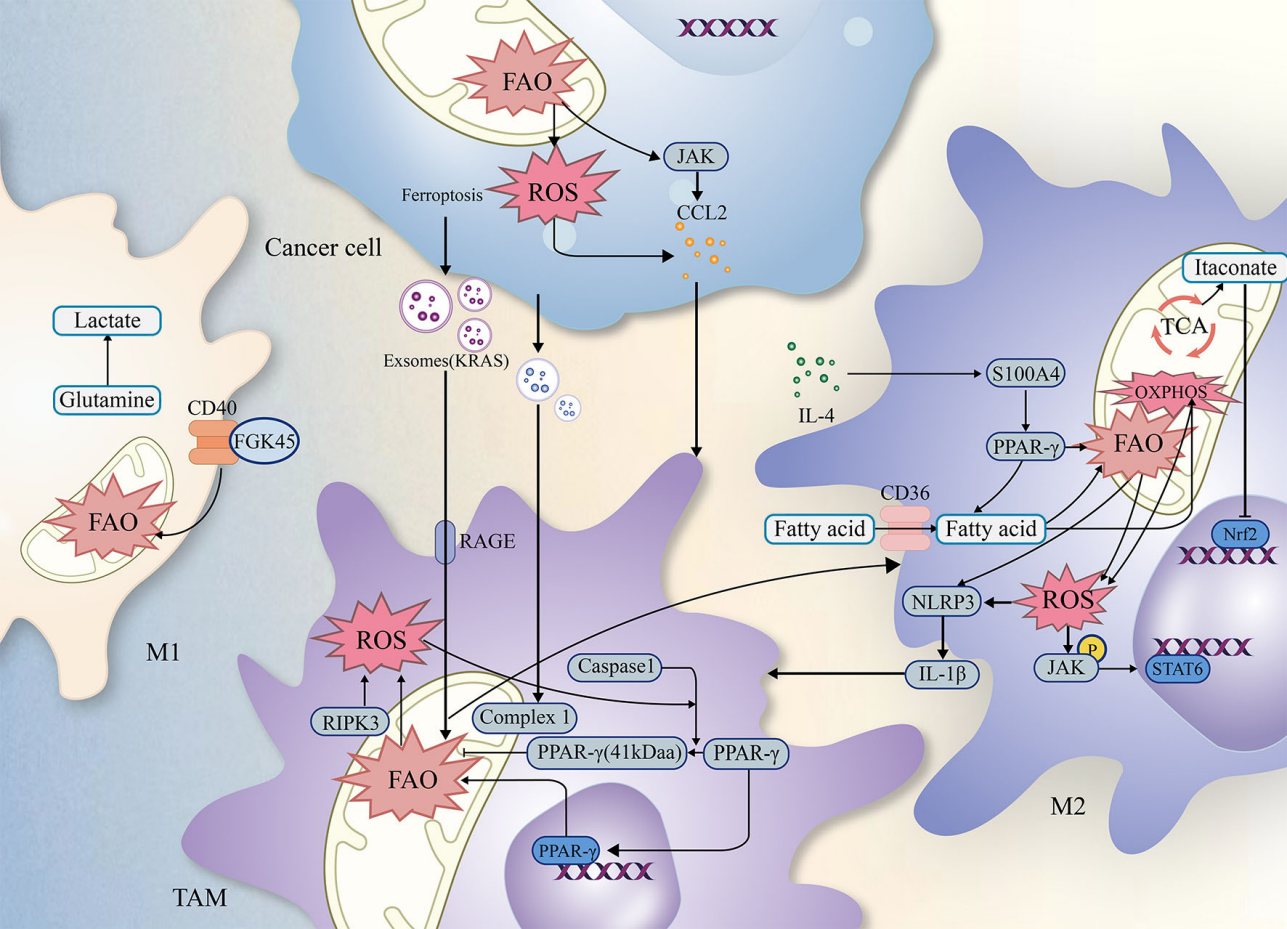
The relationship of ROS FAO and TAMs. Simplified figure depicting various possible mechanisms of TAMs associated with ROS and FAO at a cellular-level affect oncogenesis and development of tumor in TME.

Lipids, including triglycerides, cholesterol, and phospholipids, play crucial roles in cellular function. Fatty acids and glycerol constitute the triglycerides. Moreover, both tumor cells and immune cells undergo lipid metabolism, including fatty acid oxidation (FAO), reprogramming to survive the harsh environment (21). TAMs, known for their high plasticity (22), exhibit differences in FAO efficiency between M1 and M2 phenotypes. The metabolic reprogramming associated with it, especially FAO, plays an essential role in regulating tumor progression (23–25), and this article will describe how FAO synergistically influences tumor progression in conjunction with TAMs (as shown in **Figure 2**).

Previous studies have revealed complex interactions among TAMs, ROS, FAO, which may influence the biological functions of tumors through multiple factors (as shown in **Figure 2**). As a result, integrating the critical signaling pathways involving TAMs, FAO, and ROS establishes a theoretical foundation and offers research implications for further investigation into tumor treatment modalities.

## 2 ROS and TAMs

In studies investigating the regulation of tumor progression by immune cells, researchers have observed that ROS at different levels can either promote or inhibit tumor growth depending on various molecular signaling pathways. The generation of ROS involves multiple mechanisms, with mitochondria being the primary site of ROS production. Electron leakage in the electron transport chain (ETC) is the primary source of ROS (26). Electron leaking from complex I and III of ETC react with O2 to generate ROS in the inner mitochondrial membrane, influenced by the redox state of the ETC, the proton dynamics and the local O2 concentration (27). ROS can also generate in other organelles, such as endoplasmic reticulum or peroxisome (17). In addition, the nicotinamide adenine dinucleotide phosphate (NADPH) oxidases (NOX) (28), xanthine oxidase (XO) (29), cytochrome p450 (30), nitric oxide synthase (NOS) are also in ROS generation (31). The generated ROS are further involved in various molecular signaling pathways that regulate tumor cell proliferation, differentiation and apoptosis (32–34).

Growing evidence demonstrating that ROS play a role in modulating the tumor microenvironment through multiple mechanisms. ROS can directly induce cell death in tumor cells (35) and also regulate tumor progression by acting on the recruitment and polarization of TAMs (36, 37). We will discuss the relationship between ROS and TAMs and summarize the current status of research on the relevant molecular signaling pathways (as shown in **Table 1**).

**Table 1:** Targets of ROS associated with TAMs.

Target	Cells affected	Molecular/signaling events	Cellular/system events	Ref.
Recruitment				
mtDNA	Cancer cells	Damaged mtDNA activated TLR9 and induced CCL2 through NF-kB pathway	TAMs recruitment	(38)
PHD	TAMs	HIF-1alpha interacted with PKM2 *via* PHD inhibition	TAMs recruitment; induced aerobic glycolysis of M1 and transcription of IL-1β	(39, 40)
MnSOD	Cancer cells	MCT-1/IL-6/Nrf2/MnSOD	TAMs recruitment	(41)
RORalpha	Cancer cells	ROS and cytokine(IL-6, IL-8 et al) was inhibit by RORalpha *via* targeted NDUFS6 and NDUFA11	Inhibition of TAMs recruitment	(42)
Polarization				
mtDNA	Cancer cells	MtDNA released from mitochondria activated TLR9 and induced CCL2 through NF-kB pathway	M2 polarization	(38)
Inflammatory cytokines	Cancer cells	Cancer cells secreted TGF-β、IL-6、IL-13 and VEGF-A *via* ROS	M2 polarization; activated EMT and angiogenesis	(43)
PL-PUFA(PE)	TAMs	GPX4 failured to redox ROS, PL-PUFA(PE) was oxidized to PL-PUFA(PE)-OOH,and PL-PUFA(PE)-OOH was accumulated	Programmed Cell Death of TAMs	(44)
Nrf2	TAMs	Nrf2 promoted the transcription of antioxidant protein and enzymes	Inhibition of TAMs-related ROS production and inflammation	(45–47)
GM-CSF	T cell	Magmas induced GM-CSF and inhibited Caspases3/7 activation by regulating ROS levels	M1 polarization	(48–50)
MnSOD	Cancer cells	MCT-1/IL-6/Nrf2/MnSOD	M2 polarization; suppressed M1 macrophage phagocyticity	(41)
RORalpha	Cancer cells	RORalpha targeted NDUFS6 and NDUFA11, to inhibit ROS and cytokine(IL-6, IL-8 et al) production	M1 polarization	(42)

### 2.1 Recruitment of TAMs

ROS are involved in regulating the infiltration of TAMs through a mechanism of action related to influencing the macrophage recruitment, which is one of the primary ways to affect TAMs. In related studies, by activating T cells and natural killer (NK) cells, ROS recruits both neutrophils and macrophages into the TME and, in this way, kills cancer cells and inhibits tumor progression (51, 52); TAMs can also secrete ROS after being recruited to the TME, which contributes to reduces the activity of T cells and NK cells (53). Excessive ROS can damage mitochondrial DNA (mtDNA). Mitochondria of hepatocellular carcinoma cells release mtDNA into the cytoplasm in response to ROS, activating Toll-like receptors (TLRs), of which TLR9 can induce CCL2 to recruit macrophages to the TME.Additionally, TLR9 activation can also contribute to the maintenance of the M2 phenotype of TAMs (38).

In the M1 phenotype of TAMs, *via* inhibiting the prolyl hydroxylase domain (PHD), ROS-induced the generation of hypoxia-inducible factor 1alpha (HIF-1alpha). HIF-1alpha can interact with pyruvate kinase M2 (PKM2), increasing the transcriptional levels of macrophage glycolysis-related enzymes and sustaining aerobic glycolysis. It can also induce angiogenesis and participate in the recruitment of TAMs (39, 40).

### 2.2 Polarization of TAMs

The recruitment of TAMs provides favourable conditions for tumor development. As research has progressed, the interconversion mechanism of between M1 and M2 phenotypes of macrophages, involving ROS, has been recognized as a critical factor in regulating the function of TAMs. In most cases, elevated ROS promotes M2 polarization, a process in which ROS often plays a pro-tumor role. Mitochondrial Lon, a chaperonin, can induce ROS production and participate in M2 polarization, while M2 can induce Lon production, forming a positive feedback loop (43, 54). In a study of specific mechanisms, researchers found Lon can release ROS-dependent inflammatory cytokines such as TGF-beta, IL-6, IL-13, and vascular endothelial-derived growth factor-A (VEGF-A) through p38 and NF-κB signaling pathways to promote epithelial-mesenchymal transition (EMT), angiogenesis, and M2 polarization (43). In addition to the passive TAMs regulation by ROS, TAMs actively modulate ROS levels through NOX2, leading to high levels of ROS production. This, in turn, recruits and regulates other immune cells in the TME, including myeloid-derived suppressor cells (MDSCs) and regulatory T cells (Treg). Together, TAMs and these immune cells work to effectively regulate the immune function of TME, establishing an immunosuppressive microenvironment (55–59).

High levels of ROS can also limit tumor progression by activating cell death pathways. In a 2012 paper by Brent R. Stockwell, it was described that cells can Interact with excess ROS through mechanisms such as the Fenton reaction. The combination of excess ROS with a dysregulated antioxidant system resulting in the reaction of intracellular lipids with ROS, producing lipid peroxide (LOOH). And the accumulation of LOOH drives a novel form of programmed cell death called ferroptosis (60–64), which also occurs in TAMs (44). Extracellular vesicles (EVs) are also essential participants in the mechanisms involved. These exosomes can carry immunosuppressive components and chemokines (65). TAMs exposed to tumor cell-derived exosomes exhibit a metabolic profile similar to the M2 phenotype, enhancing FAO, oxidative phosphorylation (OXPHOS), and oxygen consumption rates (66–68). EVs can also down-regulate T cell immune function by inducing TAMs to produce IFN-beta and IL-6. In this way, EVs disrupt the immune function of immune cells (65).

Mammals have evolved various complex antioxidant systems *in vivo* to scavenge ROS and mitigate the harmful effects of oxidative stress, including superoxide dismutase (SOD) (69), catalase (CAT) (70), peroxidase (PRDX) (71, 72), glutathione peroxidase (GPX) (48), and mitochondrial autophagy (73, 74).Additionally, a central regulator of antioxidant genes known as Nrf2 can be dissociated from (KEAP1) under conditions of oxidative stress to exert antioxidant functions (45–47). Recently research has shown that beta-glucan with antioxidant properties can modulate ROS production in LPS-induced RAW264.7 mouse macrophages. Beta-glucan achieves this by regulating Nrf2 through the activation of the scavenger receptor Dectin-1 (75). Activation of Dectin-1 enhances the expression of the antioxidant enzyme heme oxygenase-1 (HO-1) within macrophages, thereby reducing ROS levels and oxidative stress (76). In studying the relationship between GPX and macrophages, researchers observed that dysfunctional GPX4 induced ferroptosis in macrophages by accumulating lipid peroxides (63, 64). In addition, the mammalian protein translocation mechanism, Magmas, can be involved in regulating ROS levels to maintain redox homeostasis, in which Magmas protects cells with oxidative stress damage by inhibiting the activation of Caspases3/7 (48), induces granulocyte-macrophage colony-stimulating factor (GM-CSF) to promote M1 polarization, and enhances the antigen-presenting effect of macrophage (49, 50). When targeting Mn-superoxide dismutase (MnSOD) or MnSOD-related genes to inhibit MnSOD levels, researchers observed that TAMs infiltration and M2 polarization processes were inhibited (41, 48). Retinoid orphan nuclear receptor alpha (RORalpha), which is also involved in the complex process of the antioxidant system, can significantly (42) reduce ROS levels, decreases macrophage infiltration, and enhances M1 polarization (42). All of these antioxidant mechanisms involved in the regulation of TAMs further reveal the feasibility of targeting the anti-ROS oxidative system to impede tumor progression.

To date, we have identified several tumor molecular signaling pathways regarding how ROS affects phenotypes of TAMs (as shown in **Table 1**). Directly targeting these pathways to reduce the recruitment and function of TAMs in TME and reversing M2-like TAMs to M1,or modulating macrophages phagocytosis has emerged as an extremely promising strategy for antitumor immunotherapy.

## 3 Fatty acid oxidation and TAMs

The researchers observed that TAMs predominantly exhibited an M1 phenotype during the initiation stage of tumor development. As the tumor progressed to an advanced stage, TAMs primarily expressed an M2 phenotype (21, 77). In the early stage of tumor development, TAMs preferentially utilize glycolysis metabolism for energy generation (78). However, as the tumor progressed, FAO and OXPHOS gradually became the predominant modes of TAMs’ metabolism (79). The metabolic reprogramming from M0 to M1 is achieved by inhibiting macrophage mitochondrial function and improving substrate utilization, regulated by HIF-1α and its downstream proteins (80–82). Conversely, when macrophages transitioned from M0 and M1 phenotypes to M2 phenotypes, their FAO and OXPHOS metabolic efficiency increased, accompanied by activation of the tricarboxylic acid cycle (TCA) cycle (77, 83, 84).

In studies examining the influence of lipids on tumor progression, we have observed that TAMs infiltrate more in lipid-rich droplets of TME. Furthermore, a short-term high-fat diet can activate the macrophages in adipose tissue of patients with colorectal cancer, and reduce the risk of cancer metastasis, tentatively suggesting a potential correlation between lipid metabolism and TAMs (85). Examination of the lipid metabolic profile of TAMs has revealed heterogeneity in their fatty acid metabolic profile under different phenotypes, leading to the speculation that FAO metabolic activity is associated with the TAMs’ phenotypes (77, 83). In most cases, cells undergo three steps from fatty acid oxidation to the final production of energy: FAO, conversion of acetyl-coenzyme A (Acetyl-CoA) by TCA, and OXPHOS (86). Beta-oxidation of fatty acids is the primary metabolic pathway of FAO, and when the efficiency of beta-oxidation metabolism is enhanced in TAMs, tumor-invasive abilities become stronger (87).

Further study of specific mechanisms, researchers found that the metabolic efficiency of FAO plays a crucial role in regulating mitochondrial function and polarization of TAMs. Beta-oxidation is closely related to the phenotype of TAMs (77, 83). Peroxisome proliferator-activated receptor (PPAR) system is an essential regulator of fatty acid metabolism and is involved in the metabolic reprogramming of TAMs to M2 phenotypic polarization (88, 89). The PPAR system mediated through signal transducer and activator of transcription 6 (STAT6) and PPARgamma coactivator 1-beta (PGC-1beta) elevated the metabolic efficiency of FAO in TAMs (23, 90, 91). In further studies, the expression levels of PPAR-gamma and its downstream CD36 were upregulated by the action of the upstream S100A4 protein, which induced M2 polarization responses in the form of enhanced fatty acid absorption and FAO (88). Researchers have observed that intact structure is a prerequisite for the regulation of FAO by PPARs, but receptor-interacting protein kinase 3 (RIPK3)-mediated Caspase-1 can disrupt the integrity of PPAR-gamma in TAMs, leading to the generation of a PPAR-gamma 41 kDa fragment that can move into the mitochondria. This fragment prevents the buildup of lipid droplets and the promotion of cancerous M2 cells by inhibiting the function of FAO and MCAD enzymes in a time-dependent manner (92–94). In another study, ovarian cancer stem cells can also promote the M2 polarization of TAMs with the PPARγ/NF-κB pathway (95). In addition to the PPAR system, the researchers observed that ubiquitin-specific protease14 (USP14)-mediated deubiquitination of SIRT1 can elevate its downstream fatty acid oxidation-related pathway, SIRT1/PGC-1α, in IL-4+IL-10-induced M2 macrophages. We can find the levels of M2 marker CD206 and SIRT1/PGC-1α expressed higher in USP14+ macrophages compared to USP14- macrophages. And this alteration did not affect the expression of other key proteins involved in FAO, such as PPARs, as confirmed by qRT-PCR. However, the elevated USP14 alone did not lead to M2 polarization, which further suggests that there may not be only one single FAO-related signaling pathway during the polarization schedule of TAMs (96).

Exosomes are also involved in FAO-mediated phenotypic regulation of TAMs. Kirsten rat sarcoma viral oncogene homolog (KRAS^G12D^) protein, carried by the exosome, can polarize M2-type macrophages by regulating STAT3-dependent FAO after internalized by TAMs. Moreover, macrophages with high expression of KRAS^G12D^ showed increased expression of FAO-related genes such as carnitine palmitoyl-transferase 1A (CPT1A) and acyl-CoA dehydrogenase short chain (ACADS) (97). However, unlike the results obtained by testing FAO-related genes in TAMs, the activity of the rate-limiting enzyme ACADS in TME was positively correlated with M1 and Treg infiltration levels but negatively correlated with and M2 (98). Further studies identified methylation sites of ACADs and differences in the expression of methylation levels of ACADs between cancer and normal tissues, suggesting that epigenetic alterations in ACADS may be involved in forming this phenomenon (98).

The specific signaling pathways have not been sufficiently studied. Previous studies have shown that the IFN-gamma, GM-CSF and LPS are important influences in the inducement of M1 polarization (99). Unlike the mechanisms associated with the oxidative decomposition of fatty acids alone, the secretion of IFN-gamma can inhibit the srebp1-mediated fatty acid synthesis pathway in immunosuppressed (M2-like) TAMs and stimulate FAO (100). Perhaps we can target the crosstalk between IFN-gamma and FAO to regulate the phenotype of TAMs by regulatting the secretion of IFN-gamma (101–104). However, we should not overlook the combined efficacy of the treatments. For example, inhibiting CD8+ T cells by Treg cells may enhance the secretion level of IFN-gamma, but we should also consider the anti-tumor effects of CD8+ T cells (99, 105). Therefore, a comprehensive approach is needed to select the ideal target for treatment.

In an attempt to investigate the link between metabolic reprogramming and TAMs, some metabolites are also involved in the mechanisms regulating the phenotype of TAMs (106, 107). One study found that CD40 activation altered the NAD+/NADH ratio through lactate production and enhanced M1 polarization, which relied on glutamine-lactate conversion. However, unlike lipopolysaccharide (LPS)-activated M1, CD40-activated M1 exhibited elevated activity of FAO and TCA cycle. The researchers speculate that a combination of CD40 activation and type I interferons (IFN-I) deficiency may contribute to this alteration (78, 108). Additionally, alpha-ketoglutarate (AKG), an essential intermediate in the TCA cycle, can regulate both FAO and Jumonji domain-containing protein-3(JMJD3)-dependent epigenetic modifications of the M2 genes, thereby increasing the ratio of AKG’s downstream product succinate to AKG and inducing M2 polarization (106, 109–111).

In conclusion, signaling pathways related to fatty acid metabolism exert some influence on TAMs regulation, either by directly altering FAO efficiency or by affecting the production of related metabolites (as shown in **Table 2**). The discovery of this phenomenon provides a new theoretical basis for regulating phenotypic alteration and polarization of TAMs through FAO.

**Table 2:** Targets of FAO associated with TAMs.

Target	Cells affected	Molecular/signaling events	Cellular/system events	Ref.
S100A4	TAMs	IL-4/S100A4/PPAR-γ/CD36/FAO	M2 polarization	(88)
RIPK3	TAMs	Inhibition of RIPK3 promoted FAO by regulated ROS, Caspase1 and PPARγ	M2 polarization; TAMs recruitment	(92)
PPARγ	TAMs	PPARγ/NF-κB	M2 polarization	(95)
USP14	M2	SIRT1/PGC-1α/FAO	FAO efficiency promotion	(96)
KRAS^G12D^	Cancer cells	The ferroptosis released exsomes(KRAS^G12D^) to promote FAO of TAMs *via* STAT3	M2 polarization	(97)
CD40	TAMs	CD40 promoted FAO and glutamine metabolism	M1 polarization	(108)

## 4 ROS, fatty acid oxidation and TAMs

Previous studies have demonstrated that ROS and FAO can separately regulate TAMs (38, 77, 83). For instance, modulating cellular oxidative stress levels by targeting ROS can interfere with the tumor microenvironment and modulate the phenotype of TAMs (19, 112). In addition, targeting and regulating FAO-related signaling pathways can inhibit the growth and survival of cancer cells and TAMs (79, 87, 113). In some studies, we observed that ROS could affect mitochondrial FAO (114, 115) by disrupting mitochondrial DNA (mtDNA) due to the proximity of ROS production sites to mtDNA (65, 114, 116), and such a spatial relationship provides an opportunity to study the interplay among ROS, FAO, and TAMs.

Several potential signaling pathways suggest that ROS and FAO jointly regulate the level of TAMs and tumor progression (as shown in **Table 3**). Intact mitochondrial structures support the proper functioning of FAO (38, 77, 83), and FAO-related mechanisms can regulate the phenotype of TAMs (115, 122). In contrast, NADH and FADH2 produced by FAO contribute to electron leakage processes in the ETC collectively participate in the mitochondrial generation of ROS, which further influences phenotypic changes in TAMs (123). It is due to these interconnected mechanisms that researchers have attempted to establish links between TAMs, ROS, and FAO to overcome current limitations in diagnosis and treatment.

**Table 3:** Targets involving TAMs, ROS, and FAO.

Target	Cells affected	Molecular/signaling events	Cellular/system events	Ref.
RIPK3	TAMs	RIPK3 deficiency reduced ROS production, inhibited disruption of PPAR by Caspase-1, and enabled PPAR-facilitated FAO in TAMs	M2 polarization; TAMs recruitment	(92)
CD36	TAMs	SHP1/JAK1/STAT6	M2 polarization	(24, 117)
Exosomes	TAMs	Inhibition of Complex I (NADH:ubiquinone oxidoreductase) and complex IV (cytochrome c oxidase)	M1 polarization	(118)
IL-1β	TAMs	Secretion of IL-1β regulated by modulation of FAO *via* a ROS and NLRP3-dependent manner in M2	Cancer cell Migration	(120)
CCL2	Cancer cells	Inhibition of RB promote activating of AMPK and recruitment of TAMs *via* FAO and ROS-dependent manner	TAMs recruitment	(121)
Itaconate	TAMs	Knockdown of IRG1/Itaconate inhibit FAO and ROS by reducing the efficiency of OXPHOS	M1 polarization	(119)

As mentioned, PPARs play a crucial role in regulating FAO. The deficiency of RIPK3 reduces ROS levels through the ROS/Caspase-1/PPAR pathway, which inhibits Caspase-1-mediated PPAR-gamma catabolic processes. Maintaining PPAR function and integrity improves FAO efficiency, leading to M2 polarization and TAMs recruitment (92). Scavenger receptors (SRs) are a group of endocytic receptors involved in various processes such as apoptosis, autoimmunity, inflammation, and lipid metabolism. CD36 protein is a member of SRs localized on the cell surface in adipose tissue, gastrointestinal tract, heart, skeletal muscle, and macrophages (124). In 2007, Nada A. Abumrad’s team first identified the critical role of CD36 in fatty acid uptake and lipid accumulation (125), which enables cells to generate energy through FAO instead of glycolysis (24). Current studies have revealed that CD36 can enhance the effectiveness of FAO but also promotes the generation of ROS (126). And the generated ROS levels can promote Janus kinase 1 (JAK1) phosphorylation and Src homology region 2 (SH-2) domain-containing phosphatase 1 (SHP1) dephosphorylation in response to oxidative stress, which regulates the transcription of TAM genes (24) and the polarization towards the M2 phenotype (117). Complex I (NADH: ubiquinone oxidoreductase) and III (ubiquinone: cytochrome c oxidoreductase) have been shown to have a crucial role in ROS production. Furthermore, complex IV, one of the regulatory sites of oxidative phosphorylation, is closely related to the final generation of ATP from FAO (127, 128). Complex IV (cytochrome c oxidase) expresses at higher levels in M2 compared to M1 (p < 0.05), contrasting with the difference of complex I, in which the complex I was lower in M2 than in M1 (p < 0.05) (118). The study did not reveal the specific mechanism leading to this phenomenon. However, it also enhances the possibility of the mitochondrial complexes as potential therapeutic target that involves the interplay of ROS, FAO, and TAMs. In a study on hepatocellular carcinoma cells, researchers found that changes in FAO efficiency in TAMs can regulate tumor cell migration. Further studies showed that M2-type macrophages upregulate IL-1β secretion levels by regulating FAO in a NLRP3- and ROS-dependent manner (120). The secreted IL-1β then enhances cell migration by activating the NF-κB pathway in tumor cells (129). To investigate the mechanism related to the oncogene retinoblastoma gene (RB) in malignancies, researchers knocked out the RB gene in mouse sarcoma and breast cancer models. As a result, AMPK was activated, which increased FAO by inhibiting ACC activity, thereby promoting mitochondrial ROS production and JNK activation. This activation led to the involvement of the CCL2/CCR2 axis in a mitochondrial ROS and JNK-dependent manner, recruiting immune cells, including TAMs and MDSCs, into TME (121).

In addition, some potential mechanisms that link TAMs, ROS, and FAO. Researchers have found that macrophages with the M1 phenotype are more resistant to ferroptosis compared to M2 (44), and several studies on ferroptosis suggest the interplay among TAMs, ROS, and FAO (44, 130–132). The occurrence of ferroptosis is dependent on the oxidation of ROS, and one study recently demonstrated that inducing cellular ferroptosis lead to mitochondria shrink, or even disappearance of mitochondrial ridges, impairing the function of FAO (61, 64, 115, 122). In one study, ROS and ferroptosis mediated the release of KRAS^G12D^-containing exosomes. Scavenger receptors, specifically receptor for advanced glycation end products (RAGE) on tumor-associated macrophages (TAMs), mediate the uptake of KRAS^G12D^. This uptake promotes the M2 phenotype through STAT3-dependent FAO and is positively correlated with survival rates (97).

As a critical transcription factor regulating the expression of antioxidant genes (133, 134), can reduce ROS generation through increased transcription. Nrf2 has been shown to control the efficiency of FAO by acting on OXPHOS and regulating the production of TAM-related ROS (133). Apart from its direct impact on redox processes, Nrf2 also regulates macrophages by inhibiting the transcriptional function of pro-inflammatory factors (135). In a study using itaconate to target the inhibition of KEAP1-NRF2 complex degradation, undamaged Nrf2 was released and translocated to the nucleus, activating the transcription of downstream genes. This resulted in the inhibition of both lipid peroxidation and ferroptosis in macrophages (132, 136). However, the exact mechanism of Nrf2 in TAMs still needs to be thoroughly investigated. As a macrophage-specific metabolite generated in the presence of Immunoresponsive gene 1 (IRG1), itaconate production is increased in M2 (137, 138) and correlated with beta-oxidation efficiency in TAMs (119, 139). Itaconate is one of the highly upregulated metabolites in peritoneal tissue-resident macrophages in B16 melanoma cells or ID8 ovarian cancer cells (119). Knockdown of IRG1 lead to the downregulation of itaconate and reduced FAO, OXPHOS, and ROS levels in TAMs. This significantly inhibited tumor progression, although the studies did not indicate what mechanism led to this phenomenon (119, 140). However, these influences can be considered potential clinical biomarkers while altering TAMs polarization.

## 5 The therapeutics of two targets

Considering the significance of TAMs in regulating tumor progression and the complex interactions among ROS, FAO, and TAMs, some studies have explored the inclusion of TAMs with ROS or FAO to identify more effective anti-cancer therapies (141).

Studies focusing on ROS have demonstrated that downregulation of ROS in TAMs often leads to a skewed phenotype towards M1 polarization, which provides a theoretical basis for therapeutic modalities that target NOX2, Lon proteins, RORα to alter ROS levels and thus reconfigure the phenotype of TAMs (42, 54, 56–59). In recent years, several signaling pathways between ROS and PD-L1 in immunotherapy-related studies involving TAMs have been identified (19, 56). For instance, in a triple-negative breast cancer (TNBC)-related study, induced generation of ROS in a manner that activates NF-κB signaling to promote PD-L1 expression on the surface of TAMs (142). In contrast, in the field of therapeutics combined with nanotechnology, iron oxide nanoparticles (IONPs) were found to reprogram TAMs toward an immunogenic phenotype in a manner that modulates changes in ROS production levels through the activation of Caspase-3, which is closely related to apoptosis-reduced cell survival in mouse mammary tumors (142). Additionally, researchers have observed that different levels of ROS may indicate variable tumor sensitivity to chemotherapy. Thus, it is crucial to closely monitor the dynamics of ROS during patient treatment (142). Elevated expression of FAO-related genes and increased FAO efficiency are metabolic characteristics of macrophages skewed towards the M2 phenotype (24), Inhibiting or enhancing FAO metabolic efficiency in macrophages can induce polarization towards the M1 or M2 phenotype, respectively (24). Macrophages will exhibit anti tumor effects with the M1 phenotype and promote tumors with the M2 phenotype. Therefore, shifting the balance of TAMs to the M1 phenotype by altering FAO will inhibit tumor progression. Since PPARs are critical transcription factor for FAO promotion in TAMs and is involved in regulating the polarization of TAMs, they are considered potential target for cancer therapy. In related studies, modulation of PPAR-gamma in TAMs by S100A4 and others has also emerged as a potential cancer treatment modality (88). In addition, by mediating fatty acids uptake, CD36 can regulate the metabolic efficiency of FAO and OXPHOS to influence the phenotype of TAMs. Currently, there are fewer studies specifically targeting FAO in TAMs. Most studies reprogram the phenotype of TAMs by targeting mitochondrial function or OXPHOS alone or in combination with treatment (143–145). This part of the study also provides a basis for studying FAO as a therapy target.

Previous studies show that cancer cells are susceptible to developing resistance to single treatments (146). Combining different therapeutic modalities to reduce cancer resistance and improve treatment efficacy has also been a significant challenge for researchers. Therapies that modulate the phenotype of TAMs by targeting FAO or ROS have shown effects on tumorigenesis and progression, respectively. Given the close interaction between FAO and ROS, targeting both pathways provides valuable intervention points. However, the specific mechanisms through which they influence tumorigenesis and progression are still being elucidated. Therefore, a combination therapies targeting different molecular key pathways have been selected to achieve more potent anti-cancer effects.

We found combined targeting both FAO and ROS play an crutial role in inhibiting tumor progression. Targeting RIPK3, CD36, RIPK3 can directly regulate TAMs polarization toward an anti-tumor M1 phenotype. Methods that affect CCL2 secretion (121) or the use of etomoxir and siRNA to modulate IL-1β secretion in TAMs capacity to reduce TAMs recruitment (120). Numerous therapeutic approaches targeting FAO and ROS in TAMs are currently under development. For instance, a potential therapeutic involving two targets was identified. Decitabine, a DNA methyltransferase inhibitor, was found to have an impact on the hypomethylation of RIPK3. This inhibits the FAO process in TAMs through the ROS/Caspase-1/PPAR-gamma signaling pathway and leads to a reversal of the pro-tumor phenotype of TAMs (92). An agent called α-T-K nanoemulsions, prepared with the combination of KIRA673-75 (IRE1-XBP1 inhibitor) and α-tocopherol (ROS inhibitor), had a dual inhibitory effect. Under α-T-K intervention, macrophages showed increased expression of CD86, a marker of M1-type cells, and decreased expression of CD206, a marker of M2-type cells. Simultaneously, the IRE1-XBP1 pathway, which upregulated FAO, was inhibited, resulting in decreased levels of ROS and FAO (147). This intervention led to reduced tumor cell survival and improved efficacy of immunotherapy for lung cancer. Importantly, in experimental settings, simultaneous inhibition of both ROS and FAO showed superior antitumor effects compared to either drug alone (147). And α-T-K demonstrated fewer adverse effects in a mouse model (147), so we believe this work will provide a valuable reference for cancer treatment, bringing hope for more effective tumor therapy by combining the targeting of ROS with FAO.

## 6 Conclusions

We review the signaling pathways involving ROS, FAO, and TAMs, which present new opportunities for therapeutic interventions in tumors. ROS can influence the biological function of tumors by regulating the recruitment, polarization, and phagocytosis ability of TAMs, in which ROS production and the antioxidant system *in vivo* play an important role. The efficiency of FAO metabolism and the regulation of related metabolites also impact the function of TAMs. Meanwhile, several signaling pathways that affect the biological processes of tumors have been identified, which efficiently modulate tumor progression through regulatory mechanisms involving TAMs, ROS, and FAO. However, the current research findings are still far from sufficient, and further investigations are still needed to gain a deeper understanding and explore the intricate relationship between TAMs, oxidative stress, and nutrient metabolism for potential therapeutic targets.

## Author contributions

Conceptualization, YT, LT, and ML; Investigation, LX, YT, WL, and PL; Visualization, YT and PL; Writing - Original Draft, YT, LX, and WL; Writing - Review & Editing, YT, LT, and LX; Funding Acquisition, LT and ML; Supervision, YT, WL, LT, and ML. All authors contributed to the article and approved the submitted version.
